# Recovery Capital among Migrants and Ethnic Minorities in Recovery from Problem Substance Use: An Analysis of Lived Experiences

**DOI:** 10.3390/ijerph182413025

**Published:** 2021-12-10

**Authors:** Aline Pouille, Lore Bellaert, Freya Vander Laenen, Wouter Vanderplasschen

**Affiliations:** 1 Department of Special Needs Education, Ghent University, 9000 Ghent, Belgium; lore.bellaert@ugent.be (L.B.); wouter.vanderplasschen@ugent.be (W.V.); 2 Department of Criminology, Criminal Law and Social Law, Ghent University, 9000 Ghent, Belgium; freya.vanderlaenen@ugent.be

**Keywords:** addiction, recovery, culture, migration, minority, qualitative

## Abstract

Migrants and ethnic minorities (MEM) are known to be disadvantaged concerning risk factors for problem substance use and resources to initiate and sustain recovery (i.e., recovery capital). Yet, the voices of MEM are largely overlooked in recovery literature. This study explores recovery capital through 34 semi-structured interviews with a diverse sample of MEM in recovery in two ethnically diverse cities in Belgium. A Qualitative Content Analysis using recovery capital theory allowed us to identify various recovery resources on a personal, social, and community level. While physical and human recovery resources play a central role in participants’ narratives, personal recovery capital is closely intertwined with meaningful social networks (i.e., social recovery capital) and recovery-supportive environments that maximize opportunities for building culturally sensitive recovery capital (i.e., community recovery capital). Though MEM-specific elements such as culture, migration background, stigma, and structural inequalities play a significant role in the recovery resources of MEM, the largely “universal” nature of recovery capital became clear. The narratives disclose a distinction between “essential” and “acquired” recovery capital, as well as the duality of some recovery resources. The need for developing recovery-oriented systems of care that are culturally responsive, diminish structural inequalities, and facilitate building recovery capital that is sensitive to the needs of MEM is emphasized.

## 1. Introduction

A growing share of our present-day, highly diverse society consists of migrants and ethnic minorities (MEM), defined here as persons who themselves or whose (grand)parents migrated to another country in which they reside and have a minority status [1,2]. Epidemiological studies on problem substance use among MEM highlight “the healthy migrant effect” of newly arrived migrants, who experience relatively fewer substance use problems than the majority population [3]. However, acculturative, socio-economic and minority stress experienced in the host country may lead to increased substance use rates among more settled first and other generations of MEM [4,5,6]. Hence, MEM have an increased risk of substance use problems due to physical and social stressors such as poverty, geographical and educational segregation, unemployment, acculturation difficulties, trauma and language barriers, or social exclusion [1,4]. These stressors are known to be grounded in social and structural mechanisms of inequality and discrimination. Savage and colleagues [4] point out that these risk factors are often consistently present between ethnic groups, suggesting that they are largely universal in their effects, regardless of individuals’ cultural background or migration history. While there is a long-standing tradition of investigating the epidemiology of substance use problems among MEM, as well as related risk and protective factors [1], less is known about factors contributing to addiction recovery [7]. Furthermore, research suggests the need for rich and exploratory studies concerning recovery from problem substance use across people from different MEM backgrounds [4], taking into account different ecological levels [8,9].

The new recovery paradigm [10], which increasingly dominates substance use treatment and policy across the globe, defines recovery as a dynamic, personal, and multidimensional process of change, characterized by increased wellbeing on multiple life domains [11,12,13]. It represents a shift from a clinical and abstinence-based view on addiction recovery to a strengths-based and person-centered view that looks beyond substance use [14,15]. This is also the case in Flanders (the Northern part of Belgium), where the recovery movement has gained momentum due to a recent policy shift toward more community-based mental health care and the integration of substance use and other mental health services [16]. 

Recovery refers to interactions of the person and the environment over time [13] and is facilitated by recovery capital and recovery-supportive environments and [17]. Recovery capital concerns a myriad of internal and external resources that facilitate recovery [9,18]. From an ecological point of view, recovery capital is situated on three levels. The personal level comprises physical resources (including physical health and financial assets) and human recovery capital (including personal values, skills, and knowledge). The social level encompasses meaningful relationships and social networks that are supportive of recovery, whereas the community level consists of community and cultural attitudes, policies, and resources [7,10]. 

Due to the dynamic interplay between individual conditions, social relationships, and environmental and structural factors [15,19,20], recovery capital is believed to be unequally distributed within society. This especially affects MEM populations, among whom socio-economic and health disparities are highly prevalent [8,21]. Social and structural inequities obstruct access to helpful recovery resources and increase barriers to recovery, also called “negative recovery capital”, such as poor physical or mental health and involvement in the criminal justice system [9,22]. However, a few studies have also indicated that being part of a MEM community can also facilitate recovery, through offering protective factors for problem substance use [4] and recovery capital, such as a tight cultural community, social support, cultural identity, and feelings of belonging and meaning making [8]. Anecdotal evidence and treatment demand studies suggest that MEM are underrepresented in some types of substance use treatment and often use alternative coping strategies [1].

Yet, knowledge of addiction recovery resources through the eyes of those who identify themselves as MEM remains limited, particularly in Europe [7]. The aims of this paper are therefore threefold: (1) to gain insight into the first-person perspectives of a diverse sample of MEM in recovery from problem substance use; (2) to explore personal, social, and community recovery resources that facilitate recovery (i.e., recovery capital) in this population; and (3) to elucidate barriers toward recovery. In doing so, this study offers a holistic view on recovery from problem substance use among MEM, taking into account both intrapersonal and external resources on different ecological levels [10]. 

## 2. Materials and Methods

In the study sample, we aimed to reflect the diversity of MEM in Flanders (Belgium) [23] through a purposive sampling strategy. Participants who self-identified as (1) having a migration and/or ethnic minority background and (2) being in recovery from problem substance use were recruited through venue-based methods [24] in Ghent and Antwerp (two ethnically diverse cities in Flanders). As MEM with substance use problems are considered a hard-to-reach population for research, we applied several strategies to overcome these barriers [25,26]. First, flyers were distributed at relevant venues, such as residential and outpatient drug treatment centers and low-threshold community centers for people who use substances. The first author, who also conducted the interviews, visited these recruitment sites, met potential participants, and informed them about the research [27]. Second, we enlisted gatekeepers who had a trusting relationship with potential participants to bridge the gap between the researchers and envisaged respondents. Third, a 15 euro gift voucher was offered to the respondents as compensation for their participation in the research [28]. Data collection continued until no substantially new themes were identified in the interviews and theoretical saturation was reached (*n* = 34) [29].

To gain an understanding of the recovery processes and resources of MEM, we set up a qualitative study design in collaboration with relevant service providers and practitioners who also functioned as gatekeepers. All procedures were approved by the ethics board of the Faculty of Psychology and Educational Sciences of Ghent University (ref. 2017/106). Oral and written informed consent was obtained from all participants before the interview started, in which confidentiality, anonymous reporting and the right to withdraw from this study at any time were discussed. During the interviews, the researcher was sensitive to the participants’ needs, regularly checking if they still felt comfortable by means of verbal and non-verbal cues. The semi-structured interview guideline (available upon request from the first author) was tested and revised during five pilot interviews. Interview questions focused on participants’ (1) patterns of substance use, (2) their recovery process, (3) barriers and facilitators for recovery, and (4) the role of having a MEM background in this process. The open-ended questions that were complemented with probing follow-up questions, encouraged participants to talk openly about their lived experiences, unfolding rich narrative data in which recovery resources (or the lack thereof) became clear [24]. The interviews were conducted in places chosen by the study participants, such as their home, a treatment setting, coffee bar or a public library, where they felt comfortable to disclose personal experiences. The interviews lasted between one and three and a half hours (including breaks) and were audio-taped and transcribed verbatim. 

The recovery resources that study participants encountered during their recovery processes were analyzed using Directed Qualitative Content Analysis (QCA), as described by Hsieh and Shannon [30]. Directed QCA is a concept- and data-driven method to systematically describe the meaning of qualitative data through a specific lens, enabling the integration of recovery capital theory and the lived experiences of participants. 

To distillate recovery resources from an ecological point of view, the overarching categories in the coding frame were predefined and consisted of the three levels of recovery capital, namely personal, social, and community recovery capital [11,31]. Upon familiarization with the data, the subcategories emerged through an iterative process of open (i.e., inductive coding of relevant aspects in the interviews separately) and axial coding (i.e., identifying commonalities and grouping together subthemes) [32]. To increase the validity of the research and to enhance intercoder reliability [33], the first two authors independently coded and categorized five rich interviews into a coding framework. After reaching a consensus about the coding framework in consultation with all co-authors, the first author analyzed the remaining interviews. 

## 3. Results

A sample of 34 respondents (of which 4 women), aged between 18 and 60 years old (mean: 38) participated in this study. They were mostly first- and second-generation MEM, with Belgian, foreign, or dual nationalities. At the time of the interview, self-reported problem substance use ranged from abstinence to ongoing and intermittent substance use (e.g., substitution therapy; controlled or sporadic substance use). The majority of the participants had one or multiple psychiatric diagnoses such as depression or (substance-induced) psychosis and had been in contact with specialized (outpatient and/or residential) substance use and/or mental health treatment in the past. Most of the participants did not have a high school diploma or international equivalent. The majority reported lifetime involvement in the criminal justice system. An overview of core participant characteristics is provided in Table 1. Participants were given an alias when illustrating the results with interview extracts.

**Figure 1:**
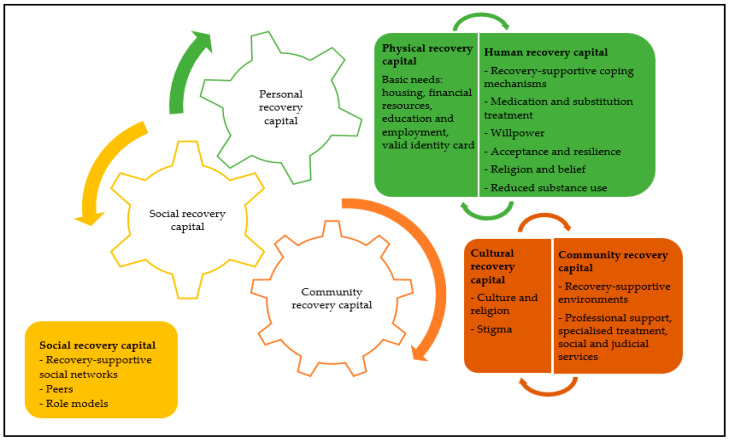
Summary of the findings.

Data analysis of the interviews uncovered various forms of personal, social, and community recovery capital that were meaningful for participants’ recovery processes. Figure 1 provides a summary of the findings.

### 3.1. Personal Recovery Capital

#### 3.1.1. Physical Recovery Capital

Basic needs, such as housing, financial resources, access to education and employment, and having a valid identity card that permits access to these resources, were considered crucial aspects of physical recovery capital. Many respondents, especially those who had experienced episodes of homelessness, described housing—more specifically, having a stable home where they feel safe—as a basic need that should be met to build other recovery resources and work toward recovery.

*(Those) Who are homeless people, who sleep on the street, that’s why they use everything. (…) That’s why he does bad things. But if somebody helped these people, I think maybe he changes his life. (…) You know why I know? Because I was on the street here.*

(Tomáš, 31 years old, #27)

Participants described how a lack of financial resources could enhance participation in criminal activities (e.g., dealing drugs), but also motivated them towards recovery when they became aware of the financial repercussions of their substance use. That is, financial resources were important to fulfill important needs, but also to pursue hopes and dreams for the future. 

The majority of the participants had low educational attainment (i.e., no high school diploma) due to early school drop-out related to mechanisms of migration, rejection, lack of parental support, substance use, diminished motivation, and deviant behavior. This decreased their chances on the job market, while many participants mentioned employment or alternative daily activities (e.g., voluntary work) as meaningful recovery resources, offering them responsibility, acknowledgment, and financial resources. 

Several participants (both first- and second-generation migrants) described how the lack of a valid Belgian identity card impeded access to recovery resources such as employment, housing, and substance use treatment.

*What’s the problem? Drugs are the problem. Let’s work on that drug problem, but that doesn’t work out because you don’t have the papers.*

(Amir, 39 years old, #22)

#### 3.1.2. Human Recovery Capital

##### Recovery-Supportive Coping Mechanisms

All participants discussed how problem substance use functioned as self-medication and coping mechanism for a variety of psychological and physical health issues, such as (childhood) trauma, depression, physical and emotional pain, sleeping problems, and craving. Even though substance use offered them a way to “forget their misery” temporarily, participants acknowledged that it was no satisfying long-term solution. Rather, they deemed it essential to address the root causes of their problem substance use, as well as to find “tools” and learn “skills” to deal with future challenges in an adaptive way. To deal with psychological issues, respondents indicated that they needed to build what can be called “emotional literacy”: processing traumatic experiences from the past and being able to constructively handle these emotions, often by sharing them with others (e.g., counselors, friends, and/or family). This was especially underscored by those who (used to) suppress feelings caused by taboos within their family and/or culture.

*You don’t feel well and then you start using to suppress your emotions. (…) I had built up walls about my past, I didn’t dare to speak about my emotions. (…) To be clean, you have to handle your problems. You have to go back to the past, where it all went wrong.*

(Abdel, 47 years old, #17)

##### Medication and Substitution Treatment

As substances were often used to self-medicate, participants described receiving medication as an important contributor to their recovery, diminishing the urge to use and enhancing their quality of life. Finding the right (dose of) medication was often considered a struggle, as it could cause side effects or turn into an addiction. In that light, participants emphasized the importance of having a doctor who listens closely to their needs and past experiences with medication. 

Several participants indicated that the use of opiate substitution treatment (methadone or buprenorphine) facilitated their ability to function “normally” in society (e.g., by going to work). However, all of them simultaneously expressed the wish to quit opiate substitution treatment, although previous attempts had sometimes resulted in relapse episodes.

*Better I drink methadone, I don’t use that shit (heroin), and I’m good. My body is good, I don’t think bad things in my mind. I relax. I take the medication. I’m happy. (…) Of course. I don’t want to drink the medication all my life. I want to stop, but slowly. (…) Methadone is okay, it helps you, but the methadone is the same like drugs, no? It kills you like drugs.*

(Tomáš, 31 years old, #27)

##### Willpower: From Necessity to Intrinsic Motivation 

The majority of the participants described recovery as an ongoing challenge or “battle” that requires willpower and perseverance.

*I have to keep gritting my teeth. The internal fight saying: no, no, no. That demands a lot of willpower.*

(Abbas, 37 years old, #3)

This willpower was often rooted in the feeling that change was necessary, due to increasing awareness of the negative consequences of substance use on participants’ physical and mental health, financial situation, social network, and overall quality of life. The contrast between “who they are with substance use” and “who they want to be” motivated them to make progress.

*This is not my life, you know my point… (…) It is no good to steal. I like to build my life, live here, have a family. I lost too much. (…) I like being again who I have been before.*

(Michal, 26 years old, #25)

Over time, intrinsic motivation grew by finding meaning, joy, and satisfaction in life without problem substance use, through meaningful activities and social networks. The majority discussed how having long-term goals motivated them in their recovery process. Many participants discussed how they desired to live a “happy”, stable, mostly substance-free life with what they considered “normal” aspects of life, such as having a fulfilling job, daily activities and structure (in contrast to the “rollercoaster” life of substance use), following education, having a home, and being surrounded by people they love. However, some also highlighted the importance of experiencing positive and exciting challenges in life, as a “boring” life was considered a trigger for relapse. Several participants also discussed how experiencing personal progress and success on multiple life domains (e.g., reduced substance use, participation in sports, or fulfilling family responsibilities) increased their self-esteem, offering them hope for the future and strength for recovery.

##### Acceptance and Resilience 

Participants described how acceptance of certain experiences was needed to move forward in life. For some, this meant accepting that the past cannot be changed. For others this was about accepting mental illness as a life companion. Accepting life as it is and being satisfied with small things was also mentioned in this perspective. Furthermore, acceptance was related to the presence of stigma in society. Stigma was mainly associated with having a MEM identity, but also with substance use, criminal records, and other intersectional characteristics. Building resilience to deal with stigma and other adversities was considered an important resource in their recovery process.

*That’s the life that I was given and it’s fucked up, but I try to make the best of it. I try to see everything positive and I know, it’s full of discrimination, racism, dishonesty, but that’s life.*

(Amir, 39 years old, #22)

##### Religion and Belief

More than half of the participants described themselves as religious (Muslim or Christian). Their faith offered them strength to cope with emotionally difficult situations and to persevere amidst challenges. By asking God to forgive past mistakes, they could find redemption and “start over”. Furthermore, the relationship between the participants and God had a social dimension, with Him “listening” to them and being a supportive figure in their recovery process. Participants cleared their minds and found meaning and understanding by reading the Bible or Koran, praying, and going to church or the mosque. Being a devoted Muslim or Christian motivated participants to work toward change because they feared being a “sinner” in the afterlife, but they also wanted to keep the promise they made to God to be(come) “clean”.

*One day I told myself: now it’s enough. I’ll take the Bible, go to church, light some candles. The book of Jesus will help me. And that’s how I lost everything (referring to substance use), little by little. Somebody asks me, you want a drag? I say no, I take the book and I read. I have to get stronger. I’m a good Catholic.*

(Miroslav, 30 years old, #16)

##### Reduced Substance Use

All participants considered abstinence or reducing their substance use as an essential, though not exclusive, part of their recovery process. Being able to reduce problem substance use could be both a result of recovery resources (i.e., facilitated by other resources) and a recovery resource in itself, as it opened up opportunities for building other forms of recovery capital.

*The longer you are sober, the stronger you are against the craving. So, sobriety is a helping element for recovery.*

(Amina, 38 years old, #24)

### 3.2. Social Recovery Capital

In some narratives, the need for “love” was strongly foregrounded. Several participants described how they had a limited social network and were highly dependent on themselves. This was related to (1) their migration background (family/friends living abroad), (2) their problem substance use (“burned bridges” within their social network by causing them harm in episodes of heavy substance use and criminality), (3) stigma and shame leading to isolation, (4) intergenerational problem substance use within the family, and (5) disconnecting from people who they considered detrimental to their recovery efforts. This contributed to participants’ feeling that they had to “do it alone”, while loneliness was also called a “pitfall for substance use”. Consequently, most respondents regarded having a recovery-supportive social network as paramount.

*The support of others means a lot. We are so used of doing it alone, but eventually you won’t do it alone. I won’t. I couldn’t get rid of my addiction alone.*

(Enzo, 27 years old, #14)

Recovery-supportive social networks consisted of family, partners, friends, and peers who were described as “understanding” and “honest” people they could “count on” if they needed help or wanted someone to talk to and listen. While (fear of) loss of social networks could be a motivator for recovery, persons who unconditionally supported their recovery journey could be so too. Several participants described a recovery-supportive social network as a “clean” network that discouraged substance use and where they could be “themselves”, without having to “wear a mask”. 

Some participants described how they went through their recovery process together with a close friend or family member in recovery, who motivated them and made them feel like they were not alone. A number of respondents found that only peers who shared substance- and/or MEM-related experiences could truly understand what they went through. These peers could also function as role models, offering participants hopeful examples regarding recovery, religious devotion, or other life domains such as sports or family responsibilities.

*That friend who took me to X (substance use treatment center) yesterday. He has the same problem as me. (…) He is actually my biggest support figure. He motivates me to go to the mosque. He tells me to stop taking heroin and stuff.*

(Kerem, 26 years old, #10)

Participants that were parents described how their children functioned as motivators in recovery, as they offered feelings of responsibility, a future-oriented perspective, and a goal in life, even when contacts were limited due to issues around child custody.

### 3.3. Community Recovery Capital 

#### 3.3.1. Recovery-Supportive Environments

All participants described the importance of recovery-supportive environments and places, such as a church or mosque, community center, riding school, gym or psychiatric hospital. These were described as places where they could unwind, tell their story, work on their skills and talents, feel welcome, find meaning and value, and meet recovery-supportive social networks with whom they could engage in social activities. For all participants, an important feature of recovery-supportive environments was the restricted availability of substances (e.g., in prison, small towns, their country of origin, or in religious communities).

*It is difficult to stop using drugs in Belgium, because you have it everywhere. And almost all your friends are on drugs. (…) In Turkey, we live in a village with 10,000 inhabitants and there are no dealers there. So if you want to smoke weed, for example, you have to drive 100 km by car. If you want to smoke heroin, you have to drive 150 km. That’s how I was able to stop.*

(Kerem, 26 years old, #10)

The narratives of the participants reveal the duality of social, cultural, and professional environments, as these environments could be considered recovery-supportive for some and as recovery-challenging for others; sometimes even at the same time. For example, while stigmatizing environments could stand in the way of recovery resources such as an understanding social network, these environments were generally characterized by limited availability and visibility of substances. Although some participants indicated that they lost all contact with their religious community due to the stigma surrounding substance use, others explained how religious community resources (such as the mosque or church) could be helpful in their recovery process, by providing them religious activities that are incompatible with substance use and offer peers, role models and meaningful goals to strive for, such as becoming a devoted Christian or Muslim. 

This duality was also mentioned in light of low-threshold outpatient drug services. While these offered meaningful professional support, participants also indicated that they stayed away or avoided peak hours due to the high availability of substances among fellow service users. Home environments often encompassed many physical and emotional triggers for problem substance use, but could also feel like “home” and surround them with meaningful people. Thus, while many participants deemed starting with a “clean slate” in a new environment to be essential for recovery, for others this would mean losing important forms of recovery capital.

*I missed everything at home. It was hard to leave everything behind, no contact with the outside world, it’s not easy. After that (admission in a therapeutic community), I was clean for a few months, but you go back to where it all started. If you put that person back in the place where he comes from, where all his user friends are, the chance for relapse is a lot bigger then when he starts over somewhere else.*

(Adil, 37 years old, #3)

#### 3.3.2. Professional Support

All participants were in contact with one or more types of professional support for help on multiple life domains. Support services that they utilized included residential or outpatient substance use and/or mental health treatment, harm reduction and social services, judicial assistance and medical care centers.

According to the respondents, recovery-supportive services were characterized by (1) valuable social interactions with unbiased professional counselors, and (2) providing access to recovery capital on multiple levels (personal, social and community). On a personal level, participants described the importance of practical (e.g., debt mediation, housing, and administrative support) and emotional support (e.g., therapy). For specialized substance use and mental health treatment specifically, participants discussed the value of long-term and residential treatment that went beyond “fixing physical dependency” and offered opportunities to reflect on the problems underlying substance use. At the social level, recovery-supportive services provided participants meaningful social networks through peers, role models and involvement of the family. At the community level, participants indicated that treatment and support should be tailored to individual needs, which requires particular attention to language, stigma, religion and cultural background and traditions. Several participants mentioned the need for professional support to help them maintain recovery outside the “safe environment” of treatment settings.

*From complete safety and follow-up to no safety at all and no follow-up, it is sometimes very difficult. (…) You overestimate yourself. (…) But you do not take into account or you do not realize that it actually happened in a safe environment and that you should not compare that with reality.*

(Muhammed, 32 years old, #31)

As more than half of the participants had been imprisoned due to substance-related criminality, they addressed the importance of offering help and treatment for substance use problems in the criminal justice system rather than merely “locking them up”. For some participants, prison lowered the threshold toward substance use and criminality, leading them to believe they “came out worse than they came in”. They explained how prison did not bring long-term solutions to their problems, due to the total lack of support and the absence of focus on the future or continuity of care after prison.

*I was in prison for three years. I was clean, but I never worked on myself, on my personality. You talk about your case and about what you did, but you don’t talk about what you want in life.*

(Abbad, 47 years old, #18)

For most respondents, however, prison was an opportunity to become abstinent and acted a stepping stone toward recovery through the decreased availability of substances (e.g., by staying on a drug-free wing), engagement in meaningful activities (e.g., voluntary work, sports), and psycho-education about substance use. Furthermore, some participants argued that detention centers could lower the threshold to treatment through providing information and referral options.

*I was in prison for thirteen months, where I was clean. And then through (social service of prison) I got in contact with a center for mental health. I saw it (prison) as an opportunity to become clean, because I thought, I can’t get this shit here. (…) It was Ramadan also, so that was an extra opportunity to quit. But it was not easy, because unfortunately, it ((drugs) is offered a lot in prison.*

(Muhammed, 32 years old, #31)

#### 3.3.3. Cultural Recovery Capital

The importance of what participants defined as “their culture” was related to (self-) identification with an ethno-cultural community and the extent to which this differed from what they perceived as “Flemish culture”. Cultural recovery capital was most apparent in the narratives of participants who self-identified with a culture that was closely tied to religion. Religious values and codes of conduct (e.g., being a “good Muslim”) had a major impact on their recovery process, both in a positive (e.g., religion and embodied religious values as recovery capital) and negative sense (e.g., secrecy and isolation due to taboo and stigma).

*Belief, culture, maintaining my values and norms, that is an important anchor in my recovery.*

(Hamid, 31 years old, #28)

Stigma and discrimination were often mentioned as impeding factors for recovery from problem substance use. Stigma was related to multiple social identities (migration background, mental health and substance use problems, criminal history, poverty,…) and discussed within the context of both general society and ethno-cultural (mainly religious) communities. Discrimination related to these multiple stigmas impeded opportunities for building personal, social and community recovery capital, for example through discrimination in education, on the job market, by judicial services and treatment providers. Several participants stated that they had to “prove themselves twice as hard” because of intersectional stigmas (e.g., stigma related to having a migration background and problem substance use).

*People look at you askew. I’m brown, that’s one thing, and then I smoke too, that’s not good huh.*

(Murat, 35 years old, #34)

Hence, participants expressed the importance of an inclusive society and community, offering equal opportunities to all persons and “looking beyond past mistakes”.

## 4. Discussion

Based on 34 in-depth interviews with persons with a migrant and ethnic minority background in recovery, this study uncovered the types of recovery capital that play a role in the recovery processes of MEM, how these support and hinder recovery, as well as how being part of a MEM group impacts recovery capital in positive and negative ways. 

### 4.1. Recovery Capital and Barriers Experienced by MEM

Physical recovery capital as an essential element to initiate and sustain recovery [15,34,35] might be especially important as MEM are more prone to have a lower socio-economic status and unstable housing situations compared to the majority population [36,37]. Barriers to recovery included limited financial resources, unstable housing situations, and lack of a valid identity card (e.g., through administrative discrimination). The importance of safe home environments can be linked to Brown and colleagues’ [38] emphasis on physical and emotional safety as the first task of trauma-sensitive recovery. Trauma was mainly related to childhood experiences of neglect and abuse (both in Belgium and in the country of origin) and was aggravated by substance use-related traumas [13].

Referring to human recovery capital, participants described being able to process and cope with trauma as an important part of the recovery process [12]. “Emotional literacy”, the capacity to understand and process emotions constructively [39], was especially important for those who identified their culture as highly stigmatizing for substance use and mental health issues and had learned not to share emotional difficulties to preserve the (family) honor [40,41]. To work on emotional literacy in treatment, language barriers can form an additional challenge for MEM [42]. The significant role of religion and spirituality in the recovery processes of MEM has been addressed in previous research [43,44,45,46]. Not all participants considered themselves religious, but for those who did, religion and spirituality took a central role in providing recovery capital and guiding recovery [47,48]. Most participants emphasized the role of internal motivation, willpower, and perseverance [20]. These were influenced by both negative experiences of “hitting rock bottom” [49] and hope and optimism for the future through interrelated positive experiences of joy, meaning, satisfaction, developing (new) social identities, and having long-term goals [44,50,51,52]. However, some participants expressed difficulties in maintaining motivation while facing issues of stigma and discrimination in society. Resilience and acceptance were identified as important resources to deal with these adversities [13,53]. Accepting what one can and cannot change as a meaningful form of recovery capital is in line with Anthony’s original view on mental health recovery [54] and the 12-step philosophy [55], though none of the respondents participated in 12-step programs. The lack of 12-step involvement among study participants is consistent with literature reporting unique barriers toward mutual aid groups for MEM, often related to cultural differences [56]. Several authors have pointed out the importance of cultural sensitivity in mutual aid groups to overcome these barriers [7,57]. 

Various personal recovery resources were rooted in social and community recovery capital. On a social level, participants identified a loving, recovery-supportive social network as an essential aspect for recovery [20,58]. Meaningful relationships, peers, and role models increased participants’ motivation to change [12], especially when shared experiences facilitate identification with these significant others [26]. Thus, social recovery capital often functions as a bridge towards other types of recovery capital and can therefore be a driving force within recovery processes [47,59,60]. However, among MEM with problem substance use, developing a recovery-supportive social network can be challenging due to encounters with stigma and discrimination, as well as migration- and substance use-related experiences of loss [7,61]. 

At the community level, we discussed the diversity and features of recovery-supportive environments, as well as the duality of some of these enabling places [62]. Illustrative is the dual role of prison, which offered opportunities to abstain from problem substance use for some, but triggered use for others. As most participants experienced complex support needs across different life domains, they appealed to a range of support services in helping them build recovery capital and work toward recovery. However, gaps between community support services and respondents’ personal context can have a detrimental influence on recovery processes [10,63]. 

MEM have been described to encounter stigma related to MEM characteristics in general (e.g., due to skin color, language barriers, cultural differences), alongside stigma related to problem substance use in both mainstream society and ethno-cultural communities [64]. While the first may result in experiences of injustice and unequal access to recovery capital [35], the latter may limit access to recovery resources such as religion (less access to religious communities), coping mechanisms, social networks, and treatment services. However, cultural communities where substance use is condemned, can also be helpful for recovery as they motivate MEM to abstain from substance use and offer a drug-free environment where the temptation for substance use is limited [7]. Besides stigma related to problem substance use and migration background, participants identified other stigmatized characteristics, such as mental health problems and a criminal history. Their narratives uncovered how different stigmas can be intersectionally related and aggravate their impact on recovery resources [65]. Concerning “ethno-cultural” facilitators, a close relationship between religion and culture as recovery facilitators became clear [66]. 

From a theoretical stance, the overarching categories of personal (both physical and human), social and community (including cultural) recovery capital [11,31] were useful in assessing helping and hindering elements in the recovery processes of MEM. Even though MEM-specific elements became apparent when delving into the subthemes of recovery capital, these subthemes showed substantial overlap with the general recovery literature [31]. We identified several needs that can be considered “universal”, such as the need for safety, connectedness and belonging, self-esteem and self-actualization [49,67]. 

**Figure 2:**
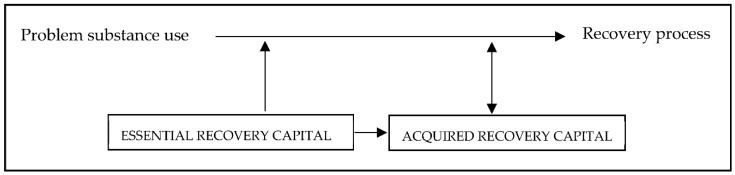
Essential and acquired recovery capital.

Finally, we distinguish between what we call “essential recovery capital” (i.e., what is considered essential to participants to initiate recovery) and “acquired recovery capital” (i.e., recovery capital acquired throughout the recovery process that also facilitates further recovery). For example, participants who felt like their physiological and safety needs were not fulfilled, experienced that meeting these needs was an essential first step towards recovery, while others placed more emphasis on establishing feelings of belonging and self-actualization [67]. When “essential recovery capital” needs were fulfilled, other needs could be realized. Acquired recovery capital is dependent on other forms of recovery capital, facilitates recovery and the building of recovery capital, and increases gradually during the recovery process. Hence, when supporting recovery, what clients mark as essential to their recovery should be addressed first and “acquired recovery capital” is likely to follow (See Figure 2). 

### 4.2. The Interwovenness of Different Forms of Recovery Capital 

While a majority of the participants originated from what is called “collectivist cultures” [59], many underscored personal recovery and the notion of “doing it themselves” as central in their recovery process. Lack of social recovery capital, due to migration- and substance use-related losses or mechanisms of social exclusion [61], can affect individual notions of recovery as a personal responsibility [10]. When closely analyzing the narratives, however, it appeared that recovery was facilitated through the interaction of distinct forms of recovery capital on different ecological levels. Religion, for example, a personal recovery resource occupying a central role in several participants’ recovery processes, was facilitated by community (e.g., religious writings, communities, and environments) and social (e.g., religious family, friends, peers, and role models) resources, but could also pave the way to these resources on social and community level [46]. This finding illustrates that a complex network of interrelated internal and external, human and non-human actors can be at play in recovery processes, supporting what Savic and Bathish [68] refer to as an actor-network approach. These authors criticize the overemphasis of personal recovery on individual agency and strengths, as it ignores the contexts that shape and constrain capacities for change, which is especially important among populations that are structurally deprived such as MEM [1]. Similarly, other authors have critiqued how neoliberal notions of individual autonomy neglect the responsibility of society in building socially accepting and recovery ready communities [10,69,70]. The Culturally Responsive Model of Recovery counters this individualistic view by positing that persons exist in a relational web comprised by family, community and larger socio-political units [59]. Consequently, MEM-related experiences of stress, social marginalization, and stigma should be integrated as socio-structural aspects central to recovery rather than obscuring these by individual notions of resilience [65]. As Price-Robertson and colleagues [59] (p. 10) assert: “Do we really want our mental health models to implicitly tell the people of these communities that the solution to their suffering lies in the space between their ears?”.

### 4.3. Limitations and Further Directions 

While this research provides valuable insights into the recovery processes of MEM, several limitations should be acknowledged. Though various strategies were implemented to minimize bias in both sampling and data analysis procedures, the findings could have been influenced by researcher, selection and response bias [27,71,72]. As the researchers may have embodied what can be regarded the “majority population” by study participants, this may have influenced who was willing to participate in this study, what they shared, and how the data were interpreted [73]. To minimize selection bias resulting from gatekeeper and venue-based sampling, we used snowball sampling as an additional sampling strategy. Yet, this yielded only two additional participants. Given the heterogeneity of MEM populations, as well as the qualitative study design, certain voices are not represented in this study (e.g., refugees and MEM with an Asian background). As only four women took part in this study, insight into the gendered nature of these experiences is also limited [52]. Interviews were conducted in Dutch, English, and French, excluding MEM that were not proficient in these languages. 

The sample allowed us to uncover shared mechanisms and experiences that are related to the personal and societal categorization of MEM and that reflect the diverse population of service users in substance use treatment [7,74]. A comparative analysis of the interviews demonstrated few particularities among subpopulations, while within-group variations and cross-cultural commonalities appeared to be more important. However, this can partly be explained by the diverse sample, as research has shown that studies including heterogeneous samples are more likely to uncover generalizable phenomena, while more homogeneous samples are more prone to offer specific input on certain subpopulations. Furthermore, some aspects of recovery capital might have been more or less prominent due to the profiles of the participants. For example, as more than half of the participants had a criminal history, it was not surprising that experiences with judicial services were eminent in the narratives. 

More research is needed to uncover intersectional mechanisms at play in the long-term recovery processes of MEM, as well as how policy and practice can promote recovery [31]. To map and identify structural inequalities, quantitative studies could broaden our knowledge on how recovery capital is distributed within society and impacted by MEM categorizations [9,64]. Furthermore, the link between problem substance use, recovery (capital), discrimination, and stigma should be investigated more thoroughly [65]. 

While some authors argue for targeted, culturally competent treatment interventions for specific groups of MEM, this approach is inadequate in addressing the complex needs of MEM [75]. As few particularities were identified between subgroups and several elements of recovery capital overlapped with the broader recovery literature, we support the idea that recovery-supportive elements align to the “universal” needs of human beings [76]. However, treatment and support services should also take into account MEM-specific experiences related to culture, migration, stigma, and trauma [77]. Macro-structural inequalities imply that MEM are often socially and economically vulnerable persons with diverse support needs and limited access to recovery capital [78]. Accordingly, professional support and policy should focus on building recovery-oriented systems of care that facilitate the development of (essential) personal, social and community recovery capital [17,20,47]. Yet, the unequal access to recovery resources should also be addressed by authorities on a macro-level by tackling stigmatizing attitudes and representations, as well as fighting discriminatory practices in policies, companies, organizations and by human beings in general [65]. 

Recovery-oriented support should be transdisciplinary and rooted in the individual needs of MEM as human beings [67]. However, to increase access to recovery capital, targeted interventions on societal, community, and personal level that decrease barriers to recovery capital are still warranted [7]. Furthermore, providing (culturally) appropriate recovery support also requires critically investigating our Westernized support cultures and adapting these services better to the needs of MEM [75]. 

## 5. Conclusions

In analyzing the experiences of 34 MEM in recovery from problem substance use, we identified several forms of personal, social and community recovery capital that overlap with the general recovery literature. Yet, when zooming in on these resources, specific MEM-related experiences of loss, stigma, and socio-structural inequalities became clear. We addressed the interrelatedness of the different types of recovery capital, as well as the distinction between what the participants considered “essential” for recovery (essential recovery capital) and what recovery capital they had acquired throughout their recovery process (acquired recovery capital). When supporting recovery among MEM, counselors and other support workers should bear in mind that recovery capital is universal to a large extent, but that MEM-specific aspects could also be of importance. To determine what kinds of recovery capital should be addressed, listening to the voices of MEM themselves is paramount. Finally, to ensure equal access to recovery capital, macro-structural inequalities that impede opportunities for MEM should be reduced by tackling manifest and hidden discriminatory practices at the macro-, meso- and micro-scales. 

## Figures and Tables

**Table 1:** Participants’ characteristics.

Nr. of Respondent (Indicated by # in Text)	Country of Origin	Gender	Age	1st-, 2nd- or 3rd-Generation MEM (Age at Immigration)	Self-Identified Problem Substance (s)	Current Self-Identified Substance Use
1	Haïti	F	36	1st (4)	Cannabis Cocaine	Sporadic cocaine use
2	Slovakia	M	32	1st (26)	Alcohol	None
3	Morocco	M	37	2nd	Cocaine	None
4	Algeria	M	43	2nd	Heroin (Methadone)	Controlled methadone use
5	Morocco	M	41	1st (23)	Alcohol Cocaine	None
6	Burundi	M	29	1st (4)	Alcohol Cannabis Cocaine	None
7	Turkey	M	40	2nd	MDMA Cocaine	Sporadic cocaine use
8	Russia	M	33	1st (16)	Alcohol Heroin (Methadone)	Non-problematic alcohol use Controlled methadone use
9	Italy	M	51	1st (11)	Heroin (Methadone)	Controlled methadone use
10	Turkey	M	26	2nd	Alcohol Cannabis Heroin Benzodiazepines (Methadone) (Suboxone)	Sporadic cannabis use Regular poly-substance use
11	Algeria	M	38	2nd	Amphetamines Cocaine Heroin	None
12	England	M	53	1st (27)	Alcohol Cannabis	Controlled cannabis use
13	Rwanda	M	25	1st (4)	Alcohol Cannabis	None
14	Switzerland/Italy	M	27	2nd	Cannabis Benzodiazepines Amphetamines Cocaine	None
15	Morocco	F	44	2nd	Heroin (Methadone)	Sporadic heroin use Controlled methadone use
16	Slovakia	M	30	1st (9)	Heroin (Methadone)	Controlled methadone use
17	Morocco	M	47	2nd	Cocaine	None
18	Turkey	M	41	2nd	Amphetamines	None
19	Morocco	M	39	2nd	Cannabis Alcohol Cocaine	None
20	Belgium/Algeria	M	33	2nd	Alcohol Speed GHB	None
21	Turkey	M	50	1st (7)	Heroin (Methadone)	Controlled heroin and methadone use
22	Morocco	M	39	2nd	Cocaine	None
23	Belgium/Algeria	F	60	2nd	Cocaine Heroïne (Methadone)	None
24	Belgium/Morocco	F	38	2nd	Cocaïne Benzodiazepines	None
25	Slovakia	M	26	1st (21)	Heroïn (Methadone	Controlled methadone use
26	Portugal	M	44	1st (41)	Heroïn (Methadone)	Controlled methadone use
27	Slovakia	M	31	1st (11)	Heroïn (Methadone)	Controlled methadone use
28	Morocco	M	31	1st (17)	Cannabis Alcohol Cocaine	None
29	Ireland	M	46	1st (28)	Alcohol Amphetamines Cocaine	Controlled amphetamine and sporadic alchol use
30	Turkey	M	37	2nd	Cocaine Benzodiazepines	Sporadic cannabis use
31	Morocco	M	32	2nd	Cocaine	None
32	Turkey	M	45	2nd	Heroin	Regular heroin use
33	Turkey	M	18	3rd	Cocaine Amphetamines	Regular cocaine and amphetamine use
34	Turkey	M	35	2nd	Cannabis	None

## Data Availability

The data presented in this study are available on request from the corresponding author. The data are not publicly available due to ethical and privacy reasons.
